# Modeling the relationship between malaria prevalence and insecticide-treated bed net coverage in Nigeria using a Bayesian spatial generalized linear mixed model with a Leroux prior

**DOI:** 10.4178/epih.e2021041

**Published:** 2021-06-04

**Authors:** Oluyemi A. Okunlola, Oyetunde T. Oyeyemi, Adewale F. Lukman

**Affiliations:** 1 Department of Mathematics, University of Medical Sciences, Ondo, Nigeria; 2 Department of Biological Sciences, University of Medical Sciences, Ondo, Nigeria; 3 Department of Physical Sciences, Landmark University, Omu-Aran, Nigeria

**Keywords:** Malaria transmission control, Insecticide-treated net, Spatial smoothed maps, Bayesian hierarchical regression, Nigeria

## Abstract

**OBJECTIVES:**

To evaluate malaria transmission in relation to insecticide-treated net (ITN) coverage in Nigeria.

**METHODS:**

We used an exploratory analysis approach to evaluate variation in malaria transmission in relation to ITN distribution in 1,325 Demographic and Health Survey clusters in Nigeria. A Bayesian spatial generalized linear mixed model with a Leroux conditional autoregressive prior for the random effects was used to model the spatial and contextual variation in malaria prevalence and ITN distribution after adjusting for environmental variables.

**RESULTS:**

Spatial smoothed maps showed the nationwide distribution of malaria and ITN. The distribution of ITN varied significantly across the 6 geopolitical zones (p<0.05). The North-East had the least ITN distribution (0.196±0.071), while ITN distribution was highest in the South-South (0.309±0.075). ITN coverage was also higher in rural areas (0.281±0.074) than in urban areas (0.240±0.096, p<0.05). The Bayesian hierarchical regression results showed a non-significant negative relationship between malaria prevalence and ITN coverage, but a significant spatial structured random effect and unstructured random effect. The correlates of malaria transmission included rainfall, maximum temperature, and proximity to water.

**CONCLUSIONS:**

Reduction in malaria transmission was not significantly related to ITN coverage, although much could be achieved in attempts to curtail malaria transmission through enhanced ITN coverage. A multifaceted and integrated approach to malaria control is strongly advocated.

## INTRODUCTION

Malaria remains the most important parasitic disease in terms of its public health implications. The World Health Organization reported a worldwide reduction in overall cases of malaria from 239 million to 219 million cases in 2010 [1]. However, epidemiological data on malaria in many sub-Saharan African countries in the last few years appear not to justify this claim. Nigeria, with 25% of global malaria cases, tops the list of the 95 countries of the world with current ongoing transmission [1]. Malaria mortality in Nigeria accounted for about 30% of the world total, and its associated burden relates to approximately 60% of outpatient visits to health facilities [2].

The programs put in place to reduce transmission of malaria in Nigeria include mass distribution of insecticide-treated bed nets (ITNs) and intermittent preventive treatment (IPT) with sulfadoxine–pyrimethamine (SP) during pregnancy. The parasite responsible for malaria is transmitted by female *Anopheles* mosquitoes; thus, ITN coverage is one of the most commonly used and cost-effective malaria control strategies [3]. The use of ITNs has been reported to increase the protection of people in malaria endemic zones from 29% in 2010 to 50% in 2017. A significant increase in accessibility to ITN was also reported during the same period [1]. In most sub-Saharan African countries, ITN delivery is either subsidized or given free to populations at high-risk of malaria [4].

As part of the efforts to reduce the malaria burden, the Nigerian government through the National Malaria Elimination Programme (NMEP) is scaling up interventions in line with the goals of the National Malaria Strategic Plan 2014-2020. Increasing ITN ownership coverage and awareness of ITN utilization was one of the most prioritized malaria prevention and treatment interventions by the NMEP [5]. The mixed-model method, which includes free, mass, and continuous distribution, has often been adopted for the distribution of ITNs. The latter approach relies on routine health service delivery activities such as immunization campaigns and antenatal care, school and community-based distribution, as well as distribution through commercial centers [6]. The NMEP is therefore instrumental to the significant increase (8% in 2008 to 69% in 2015) in the household ownership of at least 1 ITN between 2014 and 2016, as over 60 million ITNs were distributed [2,5,7]. Through efforts channeled towards malaria control, a country-based proper assessment of the ITN distribution pattern in relation to malaria control is necessary in order to monitor the success of the program at the national level.

In this study, we used a Bayesian spatial generalized linear mixed model (BSGLMM) with a Leroux conditional autoregressive (CAR) prior for the random effects to model the relationship between the spatial distribution of malaria and ITN coverage in Nigeria. CAR models have been adopted in several epidemiological studies relating to disease modeling and mapping [8,9]. We adopted the extension of the CAR model proposed by Leroux et al. [10] owing to its ability to build models in which the contiguous spatial units inherent in the data are not lost [11-14], while also accounting for the hierarchical characteristics of the data.

Despite years of efforts to abate malaria transmission in Nigeria, these efforts have practically yet to see the light of day owing to the paucity of useful information to plan a robust control program; furthermore, even where this information is available, it is always difficult to extract and synthesize it for planning nationwide malaria control programs. Therefore, the aim of this study was to use a BSGLMM where the random effects have a Leroux CAR prior to model the relationship between the prevalence of malaria and ITN coverage in Nigeria. This study provides useful information for the national malaria control program, as its findings would be important for planning, monitoring, evaluation, and the allocation of malaria control resources.

## MATERIALS AND METHODS

### Study area

The study was conducted in Nigeria, a West African country, located between latitudes 4°16ʹ and 13°53ʹ North and longitudes 2°40ʹ and 14°41ʹ East. The country has over 208 million inhabitants and ranks first worldwide in terms of the malaria burden. As a tropical country, Nigeria’s climatic conditions favor the development of mosquito vectors of the malaria parasite. Poor socio-cultural development, health facilities, and infrastructure (among other factors) are associated with malaria transmission in many endemic areas of the country.

### Sample design

The study used a nationally representative survey conducted by the Demographic and Health Survey (DHS) in Nigeria in 2018. A stratified 2-stage sample design was used for the survey. In the first stage, enumeration areas (EAs), commonly called “clusters,” were the primary sampling unit, while households in the selected EAs formed the second stage of sampling. The probability proportional to the EA size method was used to select 1,400 EAs, in which the EA size was the number of households in the EA. Household listing was carried out in all selected EAs, and the resulting lists of households served as a sampling frame for the selection of households in the second stage.

In the selection of the second stage, a fixed number of 30 households was selected in every cluster through equal-probability systematic sampling, resulting in a total sample size of approximately 42,000 households. The household listing was carried out using tablets, and random selection of households was carried out through a computer program. The interviewers conducted interviews only in the pre-selected households. To prevent bias, no replacements and no changes of the pre-selected households were allowed in the implementation stage [15].

Out of the selected households, one-third (14,000 households) were selected for malaria testing of children aged (6-59 months) [15].

### Malaria rapid testing and microscopy on thick blood smears

Household malaria diagnoses for the DHS in Nigeria targeted women and children. The diagnoses are usually carried out by personnel from the Ministry of Health of Nigeria. The SD Bioline Ag P.f (Standard Diagnostic Inc., Suwon, Korea) is used for the rapid diagnosis of *Plasmodium falciparum*, the causative agent of malaria. The SD Bioline Malaria Ag P.f (Standard Diagnostic Inc.) test qualitatively detects the histidine-rich protein II (HRP-II) antigen of *P. falciparum* in human whole blood. In addition to the SD Bioline Ag P.f. (Standard Diagnostic Inc.) rapid test, a thick smear was prepared on a slide for 75% of the households where malaria rapid diagnostic tests (RDTs) were performed. These blood smears were dried and packed carefully in the field, assigned barcode labels corresponding to the Biomarker Questionnaire, and then transported to the state-level laboratory, where they were stained. There were 18 designated staining sites in the states (1 site for each 2 states). The stained slides were then transferred to the Primary Testing Laboratory (ANDI Centre of Excellence for Malaria Diagnosis, Lagos University Teaching Hospital, Nigeria). Microscopy to determine malaria infection was carried out at this laboratory. External quality control was conducted on a selected proportion of the slides at the Secondary Testing Laboratory at the University of Calabar Teaching Hospital [15]. The diagnoses were stratified according to age, sex, and location.

### Spatial map

Spatial maps were produced using GeoDA version 1.14.0 and the hierarchal Bayesian regression was implemented in R version 4.0.2 using the CARBayes 5.2 function.

### Data likelihood and variable description

For this study, information on positive cases of malaria in subjects tested for malaria parasite using microscopy (or RDT results where microscopy was not available) was extracted and aggregated for the identifiable clusters, *C*={*c*_1_,…,*c_n_*}. These data were merged with the geographical dataset supplied by DHS, which housed data on ITN coverage, environmental factors and other socioeconomic factors already aggregated at the cluster level. After deleting inconsistent entries, information from the remaining 1,325 clusters was used for the study. The response variable was the prevalence of malaria, which was defined as a binomial experiment where n independent trials corresponded to the total number of people tested in each cluster while the number of people who tested positive for malaria is the number of successes. The covariates were ITN coverage, aridity, rainfall, maximum temperature, and proximity to water.

The spatial variation in the response variable was modeled as a function of the matrix of the covariates *X*=(*X*_1_,…,*X_k_*) and a spatial structure component *ϕ*=(*ϕ_k_*,…*ϕ_k_*) in a spatial generalized linear mixed model, as given in equation 1.

(1) $$Y_{k}|\mu_{k}\;\sim \;f(y_{k}|\mu_{k})\;for\;k=\;1,...,n,\;g(\mu_{k})\;=\;X_{k}^{T}\beta \;+\;\phi_{k}\;\beta \sim N(\mu_{\beta },\;\Sigma_{\beta })\;v^{2}\;\sim \;Inverse\;-\;Gamma(a,b)$$

In this model, the vector of covariates for the distinct cluster *C_k_* are represented by $X_{k}^{T}=(1,x_{k1},.....,x_{kp})$, of which the first term coincides with the intercept term while *ϕ_k_* captures the remaining spatial autocorrelation after the effect of the independent variables had been factored into the model. Furthermore, in the context of the generalized model, *Y_k_* is a member of an exponential family (in this case binomial) with a distribution *f*(*y_k_*|*μ_k_*) and a mean level of *E*(*Y_k_*)=*μ_k_*. The function *g*(.) is an invertible link function that relates the expected values of the response variable to the linear predictor, while *β*=(*β*_0_,…..,*β*_p_) are unknown regression parameters. This study adopted the logit link function with a data likelihood model given as;

(2) $$Y_{k}\sim Binomial(n_{k},\;\theta_{k})\;=\;\ln\left(\frac{\theta_{k}}{1-\theta_{k}}\right)\;=\;X_{k}^{T}\beta \;+\;\phi_{k}$$

Where *n_k_* and *θ_k_* are the number of trials in the *k*_th_ area and the probability of success in a single trial, respectively. As is customary in Bayesian analysis, a multivariate Gaussian prior with a mean *μ_β_*=0 and a diagonal variance matrix *∑_β_*=100,000 was assumed for *β*.

The attempt to factor spatial autocorrelation into the model necessitated a global CAR prior. Unlike the familiar intrinsic and Besag, York and Mollié model CAR priors proposed by Besag et al. [12], an alternative was developed [13] that captures the varying strength of spatial autocorrelation as a single set of random effects. As a special case of a Gaussian Markov random field, Leroux CAR priors can be expressed in a general form as *ϕ*~*N*(0,*T*^2^*Q*^-1^), where *Q* is a precision matrix. This matrix controls the spatial autocorrelation structure of the random effects and is based on a non-negative symmetric *n*×*n* neighborhood *W*. This is defined as a binary representation such that *w_kj_*=1 if the areal units (*C_k_*, *C_j_*) share a common border (denoted k-j), and is 0 otherwise. This specification forces (*ϕ_k_*, *ϕ_j_*) relating to geographically adjacent areas (that is, where *W_ij_*=1) to be autocorrelated, whereas the random effects relating to non-contiguous clusters (*W_ij_*=0) are conditionally independent given the values of the remaining random effects. A Leroux prior [10] is specified as a set of n univariate full conditional distributions *f*(*ϕ_k_*|*ϕ_–k_*) for *k*=1,…,*n* (where *Ø_–k_*)=(*ϕ*_1_,….*ϕ*_1–*k*_, *ϕ*_*k*+1_,….*ϕ_n_*). This can be expressed mathematically as:

(3) $$\phi_{k}|\phi_{-k}\sim N\;\{\frac{\rho \sum_{i=1}^{n}\;w_{ki}\phi_{i}}{\rho \sum_{i=1}^{n}w_{ki}+1-\rho },\;\frac{\tau^{2}}{\rho \sum_{i=1}^{n}\;w_{ki}+1-\rho }\}$$

The strength of spatial autocorrelation is measured by *ρ*, and a uniform prior is assigned for the unit interval of 0 and 1. Similarly, τ^2^ is a variance parameter that measured unstructured random effects and was assigned a uniform prior on the interval from 0 to 1,000.

Several Bayesian models were estimated using the Markov chain and Monte Carlo (MCMC) approach. The convergence of the models was ensured by varying the burn-in and the number of MCMC samples until a Geweke statistic value falling within ±1.96 was obtained for the parameters. Eventually, a chain with a burn-in of 20,000 and 100,000 MCMC iterations was found to be suitable for the models. The goodness-of-fit of the models was assessed by the deviance information criterion (DIC), given as *DIC*=$\bar{D}$+2pD, where $\bar{D}$ is the posterior deviance mean and posterior deviance (pD) is the number of effective parameters. The DIC comprises the terms that are a function of the data alone and also a measure of model complexity (pD). The general belief is that the smaller the DIC, the better the fitness of the model. Therefore, a model with a smaller DIC is always favored over other models. As a rule of thumb in model comparisons using DIC, a model with a smaller DIC was considered. All decisions on the significance of the variables included in the models were made using 95% credible intervals (CrIs), and a variable was considered to be associated with the response variable if the CrI did not contain 0.

### Environmental variables

The aridity index, which is defined as the ratio of annual precipitation to annual potential evapotranspiration, is a key parameter in drought characterization [16]. It is indexed between 0 (most arid) and 300 (most wet). The aridity covariate was updated for the periods of 2000, 2005, 2010, and 2015 using high-resolution grids obtained from the Climate Research Unit datasets [17]. Precipitation and potential evapotranspiration datasets were used to quantify the aridity index covariate. The maximum temperature was calculated from the modeled mean temperature and the modeled diurnal temperature range according to the method described by Harris et al. [17]. Proximity to water was defined as the geodesic distance to either a lake or the coastline. For this extraction we used only the lake dataset (L2) and the shoreline dataset (L1) at full resolution in the Global Self-consistent, Hierarchical, High-resolution Geography database. The datasets used were based on the World Vector Shorelines, CIA World Data Bank II, and Atlas of the Cryosphere [18]. We obtained rainfall data from the Climate Hazards Group InfraRed Precipitation with Stations, which has high temporal and spatial resolution [19].

### Ethics statement

The study made use of secondary and publicly available data.

## RESULTS

**Figure 1.**
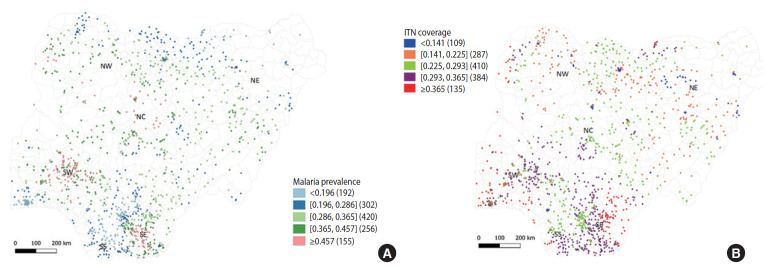
Malaria (A) and insecticide-treated net (ITN) distribution (B) across the geopolitical zones of Nigeria.

Malaria was found to be disproportionately distributed across all 1,325 clusters of the Nigerian states (Figure 1A). A stratification of malaria test results by age, sex, and the wealth quintile of children aged 6-59 months is presented in Table 1. Nationally, 36.2% and 22.6% of all children screened for malaria using RDT and microscopy, respectively, were positive for malaria. The prevalence of malaria was significantly higher in the North (NorthCentral, 0.346±0.076; North-West, 0.335±0.096) than in the South (South-East, 0.290±0.122; South-South, 0.288±0.111, p<0.05) (Table 2). However, the North-East recorded a significantly lower malaria prevalence (0.294±0.071) than the other Northern (0.335±0.096 to 0.346±0.076) and South-Western parts of Nigeria (0.335±0.157, p<0.05). ITN coverage in Nigeria is presented in Figure 1B. The distribution of ITN varied significantly across the 6 geopolitical zones (p<0.05). The North-East had the least ITN distribution (0.196±0.071) while ITN distribution was highest in the South-South (0.309±0.075).

Malaria prevalence was significantly higher in rural areas (0.338±0.098) than in urban settlements (0.287±0.121, p<0.05). ITN coverage was also higher in rural areas (0.281±0.074) than in urban areas (0.240±0.096, p<0.05) (Table 3).

Fifteen BSGLMMs were estimated. The variables included in the models, pD, and the DIC are presented in Table 4. Although the DIC estimates of models 14 and 15 were very close, model 15 was considered the best model because it had the smallest DIC value and the difference in the DIC values between the 2 models was statistically significant. Therefore, the results of the model that included all the explanatory variables presented in Table 5 were interpreted. Table 5 presents the parameter estimates of the Bayesian hierarchical regression model predicting malaria prevalence and showed that most of the covariates appeared to be significantly associated with malaria prevalence, in that their 95% CrI did not contain 0, with the exception of ITN coverage, which showed a negative and insignificant relationship. For instance, aridity (95% CrI, 0.194 to 1.507) maximum temperature (95% CrI, 3.863 to 10.522), rainfall (95% CrI, 0.176 to 1.606) and proximity to water (95% CrI, 0.236 to 0.448) had positive and significant relationships with malaria prevalence.

In addition to the covariates, the regression results showed that the estimate of the spatial correlation parameter was about 0.996, with a narrow 95% CrI (0.986 to 1.000), indicating a strong spatial dependence in cluster-level random effects. The significance of the unstructured random effect variance (τ^2^=0.130; 95% CrI, 0.106 to 0.158) also indicated that the prevalence of malaria is not the same across the clusters, as is commonly assumed in non-spatial generalized linear mixed models.

Table 6 presents the estimation results for urban and rural places of residence. It was clearly noted that there was a discrepancy in the relationship of malaria prevalence and the independent variables when the data were separately analyzed for rural and urban settings. For instance, ITN coverage was not significantly related with malaria prevalence in rural areas, though it had the expected negative sign, whereas it showed a positive and statistically significant correlation with the response variable in urban areas.

## DISCUSSION

Malaria has continued to be a public health threat despite the efforts put in place to reduce its transmission in Nigeria. There was wide variation in the distribution of ITNs across the 6 geopolitical zones in this study, but ITN coverage was not affected by location. Our model showed a negative relationship between malaria prevalence and ITN coverage for the pooled model and the rural model, but the relationship was not significant. A positive and significant association between malaria prevalence and ITN distribution in the urban model could be conceived as indicating that the reduction in malaria prevalence experienced in urban areas when compared with their rural counterparts was due to factors other than ITN distribution.

The low prevalence of malaria recorded in the North-East, especially Borno State, is similar to the findings of a previous report that Borno State was the best-performing state in terms of malaria eradication in the North-East region [20]. While the factors of sparse vegetation, scanty rivers, changes in temperature, humidity, altitude, and deforestation were implicated to have influenced malaria transmission in the area [20,21], the continuous spates of terrorism, especially in the rural communities of the state, could hamper data sourcing, thereby reducing the amount of data available for the region. The latter reason could also be responsible for the lower ITN coverage in the region. A report by the Nigeria Malaria Indicator Survey of 2010 put the North-Eastern States ahead of other Northern regions of Nigeria in term of ownership of ITNs [22], but the current data were extracted from the 2018 DHS, thus reflecting the negative impacts of terrorism and conflict on malarial control in the region. The general observation of a higher prevalence of malaria in the North than in the South is consistent with our earlier study on the incidence of malaria in relation to some environmental variables in Nigeria (2000-2015) [23]. The impact of confluent lakes and rivers in the North-Central region and the general poor health facilities in many isolated rural communities in the North have been suggested as predictors of high endemicity of malaria in the region [24]. Terrorism, which is now a usual occurrence in all the regions of Northern Nigeria (although more rampant in the North-East), could also impede malarial control programs.

Our study showed that malaria also had a higher prevalence in rural areas, an observation that is similar to a previous report from Burkina Faso [24]. Studies have indicated that rural and remote areas provide conducive environmental and climatic conditions for the development of *Anopheles* mosquitoes and malaria parasites [25-27]. Rural areas are also more likely to face challenges in terms of poor housing, poor knowledge of malaria transmission, negative health practices, and poor malaria management due to difficulties in accessing health facilities [24,28].

The negative association of ITN coverage with malaria prevalence noted in our model in this study indicated that the ITN coverage was associated with a decline in malaria prevalence across the clusters under consideration in Nigeria. However, ITN coverage has yet to exert a significant reduction in malaria transmission in Nigeria. Studies have shown that awareness and good knowledge on the utilization of long-lasting insecticide-treated nets (LLINs) and their possession may not necessarily corroborate their actual utilization [29,30]. Barriers against the utilization of ITNs or LLINs were identified as heat, reactions to the chemical, unpleasant odor, and cost [29,30]. The same reasons may contribute to the positive, but insignificant relationship between ITN coverage and the prevalence of malaria in urban areas as shown in our model.

The Bayesian hierarchical regression showed that higher ITN coverage in rural settings of Nigeria was not significantly associated with lower malaria endemicity in this study. Mapping out rural areas for ITN distribution is less cumbersome, and information dissemination could be more effective. These factors could have an impact on reaching a larger proportion of the population as part of malaria control initiative programs. However, a low-level of knowledge, insufficient health personnel, and inadequate health facilities could undermine viable malaria control programs in rural areas. Furthermore, the higher malaria prevalence in some states with higher ITN coverage could denote some degree of failure in the implementation and administration of malaria control programs. Poor coordination in ITN distribution is often noticed during mass distribution in Nigeria. Observations have shown that some members of a household might receive more ITNs than they needed, while several others might be unable to get even a single ITN. Furthermore, as earlier mentioned, having ITN may not translate to its utilization.

A comprehensive malaria transmission mitigation program is recommended to address the ineffectiveness observed in the use of ITNs in some areas in Nigeria. This more comprehensive malaria control alert system is visible in Lagos State, which is one of the areas least affected by malaria in the country. The efforts to mitigate malaria transmission in Lagos State include integration of mass distribution of LLINs into existing health services such as child immunization and antenatal care in public health facilities, indoor residual spraying (IRS) that is believed to have protected over 3 million inhabitants, larviciding of water bodies shielding over 900,000 people, and free and routine administration of SP for IPT (IPT-1, IPT-2, and IPT-3) for malaria during pregnancy [31]. Even when ITN coverage is defective, the prioritization of IRS and larviciding could make up for the deficit.

Our study showed that aridity, maximum temperature, rainfall, and proximity to water were positively correlated with malaria prevalence in Nigeria. A study from Nepal using generalized additive mixed models, however, differed from our study in terms of the relationship between rainfall/maximum temperature and malaria incidence as no significant relationships were observed [32]. While rainfall creates temporary water pockets as breeding sites for the mosquito vector of malaria parasites [33], temperature influences the development of the infective stage of the malaria parasite and the mosquito vector [34]. In Eritrea, aridity was believed to negatively influence malaria transmission [35]; however, the local adaptation of *Anopheles gambiae* (a common vector of the malaria parasite in Nigeria) to xeric habitats has been reported [36]. The latter factor may overshadow the effects of aridity in Nigeria.

Malaria is actively transmitted across the 36 states of Nigeria. The proportion of ITN coverage is still low in many endemic areas and the non-significant negative relationship between malaria transmission and ITN coverage is worrisome. To effectively harness the impact of malaria control programs, it is necessary to identify significant malaria transmission hotspots across Nigeria. Targeted deployment of scarce malaria control resources to these areas could produce a better control approach. At this point, it is crucial to evaluate the social context of adoption and utilization of ITN brands used for malaria campaigns, while also taking into consideration the all-encompassing distribution approach. Besides mass ITN distribution, the government and other stakeholders in malaria control should redouble efforts to implement other approaches. A multifaceted and integrated system is a sure-fire approach for achieving long-lasting malaria control efforts.

## Figures and Tables

**Table 1.** Prevalence of malaria in children aged 6-59 months according to background characteristics

Characteristics	Malaria prevalence according to RDT		Malaria prevalence according to microscopy	
	No. of children	RDT positive	No. of children	Microscopy positive
National	11,361	36.2	8,296	22.6
Age (mo)				
6-8	687	27.5	482	19.1
9-11	598	21.8	448	13.1
12-17	1,450	30.1	1,064	17.9
18-23	1,142	31.9	857	19.7
24-35	2,429	36.6	1,775	20.1
36-47	2,525	40.5	1,856	24.3
48-59	2,519	42.7	1,817	30.8
Sex				
Male	5,804	36.6	4,279	23.4
Female	5,547	35.7	4,019	21.8
Wealth quintile				
Lowest	2,115	57.1	1,479	38.4
Second	2,230	50.3	1,572	33.6
Middle	2,398	38.6	1,750	24.2
Forth	2,377	25.9	1,765	14.7
Highest	2,231	10.7	1,731	5.7

RDT, rapid diagnostic test (SD Bioline Ag P. f.); Children whose mothers were not listed in the household questionnaire were excluded. Source from National Population Commission. Nigeria Demographic and Health Survey 2018; 2019 [15].

**Table 2.** Malaria prevalence and ITN coverage by geopolitical zone

Variables	n	Mean±SD^1^	95% CI for mean	
			LL	UL
Malaria prevalence				
North-Central	249	0.346±0.076^a^	0.336	0.355
North-East	194	0.294±0.071^b^	0.284	0.304
North-West	275	0.335±0.096^a^	0.324	0.346
South-East	184	0.290±0.122^b^	0.273	0.308
South-South	208	0.288±0.111^b^	0.273	0.303
South-West	215	0.335±0.157^a^	0.314	0.356
ITN coverage				
North-Central	249	0.281±0.061^c^	0.273	0.288
North-East	194	0.196±0.071e	0.186	0.206
North-West	275	0.222±0.080d	0.212	0.231
South-East	184	0.297±0.072^ab^	0.287	0.308
South-South	208	0.309±0.075^a^	0.299	0.319
South-West	215	0.291±0.089^bc^	0.279	0.303

ITN, insecticide-treated net; SD, standard deviation; CI, confidence interval; LL, lower limit; UL, upper limit.

1 Mean±SD with different superscripts are significantly different, with a>b>c>d>e; Mean separation was done using the Duncan multiple range test.

**Table 3.** Malaria prevalence and ITN coverage between urban and rural areas

Variables	Residence location	n	Mean±SD	T-statistic (p-value)
Malaria prevalence	Urban	539	0.287±0.121	-8.515
	Rural	786	0.338±0.098	<0.001
ITN coverage	Urban	539	0.240±0.096	-8.687
	Rural	786	0.281±0.074	<0.001

ITN, insecticide-treated net; SD, standard deviation.

**Table 4.** pD and DIC of 15 partial pairwise spatial generalized linear model

Model	ITN	Aridity	Max Temp	Rainfall	Proximity to water	pD	DIC
1	√					358.430	6489.939
2	√	√				354.866	6483.613
3	√		√			359.214	6488.156
4	√			√		347.353	6462.155
5	√				√	342.599	6463.211
6	√	√	√			355.442	6477.694
7	√	√		√		347.604	6463.761
8	√	√				337.540	6456.714
9	√		√	√		345.145	6453.468
10	√		√		√	344.299	6461.106
11	√			√	√	330.492	6436.291
12	√	√	√	√		345.640	6454.411
13	√	√		√	√	330.882	6436.802
14	√		√	√	√	322.992	6418.675
15	√	√	√	√	√	324.069	6418.482

pD, posterior deviance; DIC, deviance information criterion; ITN, insecticide-treated bed nets; Max, maximum; Temp, temperature; √, Signifies variable(s) in the model.

**Table 5.** Bayesian hierarchical regression of the predictors of malaria prevalence

Predictors	Posterior median	95% credible interval		Geweke.diag
		2.50%	97.50%	
(Intercept)	-17.092	-23.036	-11.143	-1.1
ITN coverage	-0.061	-0.755	0.645	-0.3
Aridity	0.864	0.194	1.507	1.2
Maximum temperature	7.169	3.863	10.522	1.5
Rainfall	0.870	0.176	1.606	-1.4
Proximity to water	0.344	0.236	0.448	1.6
τ^2^	0.130	0.106	0.158	1.1
*p*	0.996	0.986	1.000	1.1

ITN, insecticide-treated net.

**Table 6.** Bayesian hierarchical regression of the predictors of malaria prevalence in rural and urban populations

Predictors	Urban			Rural		
	Estimate	2.50%	97.50%	Estimate	2.50%	97.50%
Intercept	-19.774	-28.765	-10.721	-17.092	-23.036	-11.143
ITN coverage	1.727	1.237	2.210	-0.061	-0.755	0.645
Aridity	-0.360	-1.212	0.512	0.864	0.194	1.507
Maximum temperature	6.250	1.163	11.321	7.169	3.863	10.522
Rainfall	2.413	1.426	3.389	0.870	0.176	1.606
Proximity to water	0.404	0.271	0.537	0.344	0.236	0.448
τ^2^	0.197	0.155	0.249	0.130	0.106	0.158
*p*	0.996	0.982	1.000	0.996	0.986	1.000
DIC	2663.739			3849.241		
pD	162.967			195.415		

ITN, insecticide-treated net; DIC, deviance information criterion; pD, posterior deviance.
